# *Oryzias melastigma* – an effective substitute for exotic larvicidal fishes: enhancement of its reproductive potential by supplementary feeding

**DOI:** 10.1186/2193-1801-2-235

**Published:** 2013-05-24

**Authors:** Abir Lal Dutta, Sajal Kumar Dey, Debargha Chakraborty, Asim Kumar Manna, Pankaj Kumar Manna

**Affiliations:** Department of Zoology, University of Kalyani, Nadia, Kalyani, West Bengal India; Department of Environmental Science, University of Kalyani, Nadia, Kalyani, West Bengal India; Khejuri College, Vidyasagar University, East Midnapore, West Bengal India; Academy of Biodiversity Conservation, 97, Bangur Aveneue, Calcutta, West Bengal India

**Keywords:** *Oryzias melastigma*, *Ulothrix*, Larvicidal fish, Biological control, Indigenous, Reproductive potential

## Abstract

A preliminary study was conducted on the efficacy of *Oryzias melastigma* in consuming mosquito larva so as to control mosquito and mosquito borne diseases, and enhancing its reproductive success using supplementary feed. *Oryzias melastigma* is a larvivore fish and widely distributed in the shallow water, wetlands of Gangetic plains and peninsular India. These studies indicate that *O. melastigma* is a prolific breeder and gregarious feeder of mosquito larvae. Increased reproduction by providing different supplementary feed, of which *Ulothrix* acted remarkably, may aid in wide spread use of this fish as a biological control measure against mosquitoes. One adult fish of any sex can consume 87.1% first instars mosquito larvae/day. So, early stages of mosquito larvae are effectively controlled, as compared to other successive stages. *Ulothrix* has considerable effect on egg production, successful hatching and regaining reproductive maturity of female in surprisingly quicker interval.

## Background

Mosquito borne diseases are a major problem in all tropical and subtropical countries and are responsible for causing some of the most life threatening diseases in man, like malaria, dengue fever, filariasis, encephalitis and chikungunya. The harmful effects caused by chemicals, for instance DDT, in mosquito as well as on non target populations and the development of resistances in mosquitoes have prompted an alternative use of simple and sustainable methods of mosquito control (Milam et al. 2000).

Larvivorous fishes are being successfully exploited for mosquito control in various countries like Spain, Italy, Greece, Southern Europe, Northern Africa, India, Iran, Malaysia, Madagascar and many other countries (Bruce-Chwatt 1985). They are either employed for the destruction of larvae or to render the habitat unsuitable for mosquito to breed.

Larvivorous fishes are those that feed on immature stages of mosquitoes. They are mostly small, hardy and capable of maneuvering easily in shallow waters where mosquitoes breed. They must be drought resistant and capable of flourishing in both deep and shallow waters. They must have the ability to withstand rough handling and transportation over long distances. These fishes must be prolific breeders with a shorter span of life with having the ability to breed successfully in confined waters. They should be surface feeders, carnivores in habit and should have a predisposition to feed on mosquito larva even in the presence of other food materials. Among important criterion for all larvivorous species is that they should not be brightly coloured or attractive. Besides, they should be unpalatable with no food value so that they are discarded by fish-eating people (Job 1940).

*O. melastigma* (McClelland 1839) belonging to the Order Beloniformes, Family Adrianichthyidae and Subfamily Oryzinae (Jayaram 1981), is a tiny cyprinodontid weed fish. It is a carnivorous, surface feeder found in both lentic and lotic waters. It is a semitransparent and hardy fish which can tolerate a wide range of salinity (31ppm) (Manna 1989), temperature, and many other adverse water qualities. Popularly known as rice fish or minnow (Rosen and Parenti 1981) or Indian Medaka or Bechi, it is a sexually dimorphic species (Manna and Bannerjee 1984). It is found in limited areas of West Bengal, Tamil Nadu, Kerala, Orissa (Jayaram 1981; Manna and Bannerjee 1985) in India and also some riverine areas of Bangladash. In West Bengal, they are mainly distributed in the lower Gangetic shallow water bodies of 24 pargans, Midnapore and Howrah district. They generally lay eggs two to four times in a year and show a notable parental care and their breeding rate is higher as compared to other minnows under certain conditions (Daniels and Ranjit 2002). This experiment has been designed to study the increase in reproductive success of *O. melastigma* by giving different supplementary feeds and to study its potentiality in mosquito control.

## Methods

In the present experiment *O. melastigma* were collected from fresh water pond of Midnapore and maintained in a cement cistern (20 liters), where they could breed successfully. The eggs were collected time to time, hatched in laboratory condition and reared in separate glass aquaria (55.88 cm × 30.48 cm × 30.48 cm), each filled with 20 liter of tap water (1/4th of aquarium). The hatchlings were fed with natural feed till maturity. Six months old, healthy, disease-free specimens of both sexes of first filial generation were used for the present study.

Five males and five females of above specifications were released in experimental aquaria. Five such replicas were used for each of the first to third instars larvae and for pupae. Two earthen vats were maintained for stocking of mosquito larvae collected from drains and ditches. Sieves of specific mesh sizes were used to separate the larval insters of mosquitoes. After one hour of fish release, 100 larvae of desired instars were introduced each time in each experimental aquarium at 2 hourly intervals (6 times a day) and were observed till the end of tenure of the schedule. Suction pipettes were calibrated to count different larval instars in specific numbers for introduction in each aquarium each time. The fishes were fed and observed for 12 hours daily, for 20 consecutive days and the numbers of larvae consumed were noted and the specimens left were counted and removed. During rest of the observation period (12 hours) no food was supplied to keep them for food appetite. Reasonably sufficient numbers of larvae were supplied each time to minimize their energy expenditure for food search and to increase energy budget for feeding of minnows. However, preference of different species of mosquito larvae by the juveniles and sub-adults of the *O. melastigma* was not tried.

In a separate experimental set up, five pairs of adult *O. melastigma* (6 month old) were kept in each of the six aquaria where they were provided with different kinds of food supplement such as rice, semolina (suji), *Ulothrix*, and combinations of rice and *Ulothrix*, semolina and *Ulothrix*, and, semolina, rice and *Ulothrix*. Another set of five pairs of adult fishes were fed with natural food and was used as control. Water parameters were maintained; temperature from 26°C to 31°C, pH from 6.34 to 6.61 and dissolved oxygen content from 5.28 to 6.47 mg/ml. The behavioral patterns up to three generations were critically studied.

**Figure 1:**
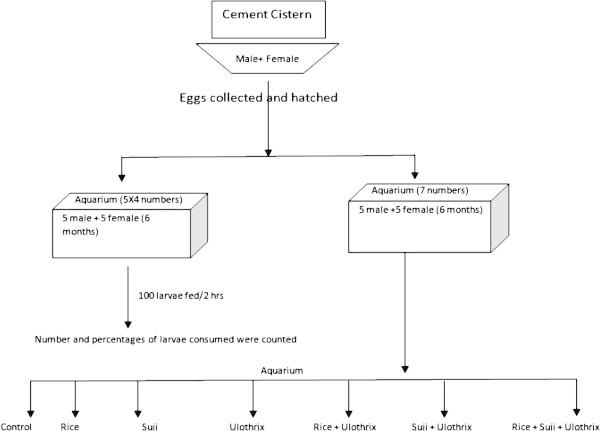
**Schematic diagram of experimental design.**

After each successful fertilization, from each of the aquaria, egg clutches were removed carefully with the help of a blunt forceps. Clutch size was measured and transferred to separate aquaria. Viability of the eggs was calculated in terms of fertilization success and hatching success. Hatchlings were reared to maturity to calculate the sex ratio of the new born. The design of present work is summerised schematically in Figure 1.

Data was statistically analysed using t-test. The maximum significant level chosen was *p < 0.05*.

## Results and discussion

**Figure 2:**
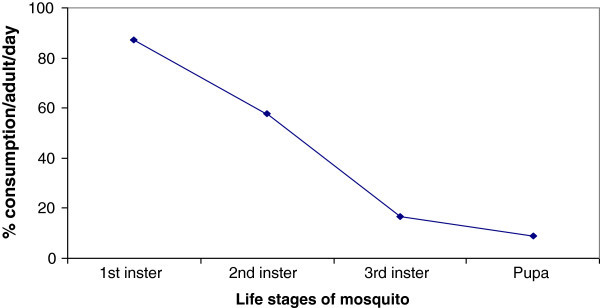
**Percent consumption of different larval stages by*O. melastigma.***

Results based on multiple observations from 1st experimental set up demonstrate that adult *O. melastigma* feed successfully and vigorously on the early larval stages of mosquito. The first instars larvae were observed to be consumed at an average above 50 numbers/day/fish. The consumption rate decreased successively towards the advanced stages of mosquito and reached below 10 numbers/day/fish for the pupae. The percent consumption of 1st, 2nd and 3rd insters and pupae by each fish/day are presented in Figure 2. Results obtained from the 2nd experimental set-up show different reproductive efficiencies in fishes provided with different supplementary feed. Fishes fed with *Ulothrix* show a marked increment in egg production, fertilization success and hatchling survival as compared to other groups, including control. While each control female produces 44.667 ± 1.196 eggs, female fed with rice and suji produces 45.25 ± 1.538 and 45.083 ± 1.554 respectively, the *Ulothrix*-fed female produces 67.083 ± 1.311 eggs per laying. When *Ulothrix* was fed in combinations with rice, suji and rice-suji mixture, the clutch size per female was observed to be 48.833 ± 1.036, 50.167 ± 1.120 and 50.083 ± 0.996 respectively. *Ulothrix*, whether fed singly or in combinations, shows reasonably significant improvement in clutch size produced per female.

When percentage of fertilized eggs from the clutches was calculated, it was observed that 60.583% of control eggs, 64.167% eggs of rice fed group, 62.917% eggs of suji fed group, 78.167% eggs of *Ulothrix* fed group, 67.333% eggs of rice + *Ulothrix* fed group, 67.583% eggs of suji + *Ulothrix* fed group and 71% eggs of suji + rice + *Ulothrix* fed group were fertilized. Hatching success, calculated as percentage of fertilized eggs hatched, shows highest viability of eggs of *Ulothrix* fed group. 95.833% of fertilized eggs hatched successfully in this group. 80.583%, 84.083% and 86.75% of fertilized eggs in control, rice fed and suji fed groups were observed to be hatched respectively. *Ulothrix* along with rice or suji or rice + suji resulted in 85.413%, 84.75% and 87.25% hatching success respectively. *Ulothrix* has shown significant increase in producing fertilized eggs, whether fed singly or in combinations. However, combinations have no significant effect in increasing hatching success (Table 1).

**Table 1:** **Results of supplemented feeding on reproductive efficiency of*O. melastigma***

	C	R	S	U	R + U	S + U	R + S + U
**Clutch size**	**44.667 ± 1.196**	**45.25 ± 1.538**	**45.083 ± 1.554**	67.083 ± 1.311**	48.833 ± 1.036*	50.167 ± 1.120**	50.083 ± 0.996**
**Fertilized eggs (%)**	**60.583 ± 1.593**	**64.167 ± 1.604**	**62.917 ± 1.721**	78.167 ± 2.584***	67.333 ± 2.426*	67.583 ± 2.454*	71 ± 1.871***
**Hatching success (%)**	**80.583 ± 1.823**	**84.083 ± 1.721**	86.75 ± 1.733*	95.833 ± 0.796***	**85.413 ± 1.900**	**84.75 ± 1.883**	87.25 ± 1.706*

All data are presented as mean ± SEM from 12 similar experiments. Data are significant at the level ***p < 0.001, **p < 0.01, *p < 0.05.

Female:male ratio was observed above 1 in both the control and *Ulothrix* fed groups. The value was calculated as 1.07 in control which significantly increased to 1.33 in *Ulothrix* fed group. Removal of egg clutches from female enhances the reproductive maturity effectively. Adult females spawn biannually in the wild while the removal of eggs causes them to produce eggs 4 times a year in all the experimental groups except the *Ulothrix* fed group where it is found to increase phenomenally to 4 times a month i.e. 48 times a year. The higher ratio of female offspring and shortening of gestation period in *Ulothrix* fed group caused a dramatic rise in per annum production by a single pair of *O. melastigma.* It was calculated that a pair of control fish and their successive generations may cause a total production of about 4 Kg per year whereas an *Ulothrix* fed pair and their forerunners would add up to a quintal per year.

Mosquitoes, potent vector of various life threatening diseases, are and will be the major concerns for human health. To find an effective solution for their control in the wild calls for introduction of efficient larvivorous fishes which prey on them in their natural habitat. A variety of larvivore fishes were tried and established as biological control measure against mosquitoes, of which most are exotic to Indian water bodies. For instance *Gambusia affinis* and *Poecilia (Lebistes) reticulate* have been extensively used in India for mosquito control. Though they have been found to be efficient in mosquito control, their adverse impact on local biodiversity must draw some attention of the ecologists. It has been reported that where *Gambusia* was introduced, often for mosquito control, has resulted in or has contributed to the exclusion of many native fishes which have similar ecological requirements (Page and Burr 1991). From a conservational view point, to protect the habitat of native species, World Health Organization has emphasized on research and introduction of indigenous fish species for mosquito control. The subject of present study, *Oryzias melastigma*, is a native species to Indian subcontinent distributed widely in many states of India and Bangladesh and is a hardy fish capable of propagating in shallow natural water bodies which happen to be the breeding ground of mosquitoes. From the present study it has been found that this fish has reasonably high feeding activity on mosquito larvae, especially on the early stages of mosquito life cycle. The efficiency of consumption gradually decreases as the life stages of mosquito progresses. This may be due to that the mouth of fish appears to be non-accommodative to engulf smoothly the larger larvae. Besides, as the larva develops, they sense the water movement more and learn to move away from the predator faster. It is also expected that the wriggling movement of the larvae is more attractive than the comparatively stationary late larvae and pupae.

The vigorous larvivore activity of this fish, as advocated in the present study, may suggest promote the rearing and breeding of *O. melastigma* for the control of mosquitoes. It also can be suggested that this fish is an efficient native alternative to the exotic vector control agents like *Gambusia affinis* and *Poecilia (Lebistes) reticulate*, as far as the larvae feeding ability is concerned (Chatterjee and Chandra 1997a, b).

Breeding of *O. melastigma* in captive condition has shown encouraging results with supplementary feed. It is found that the fish can be readily cultivated under laboratory conditions with different feed supplement exhibiting varying degrees of reproductive success. *Ulothrix* was observed to be most promising in enhancing the egg production, fertilization success and egg viability when supplemented singly or in combinations. *Ulothrix* must have some inducing effect on gonadal activity of both male and female fishes which need to be studied further. Results show that female to male ratio of produced offspring is on higher side in *Ulothrix*-fed pairs as compared to that of control pairs. Furthermore, removal of egg clutches from the female fed with *Ulothrix* resulted in an enhanced reproductive maturity leading to a remarkably high egg producing ability. All these reproduction enhancements aid in huge production of *O. melastigma* seeds that can be successfully released in the mosquito breeding grounds like drains/canals, septic tanks, cement tanks, pools/ponds, pit latrines, marshy lands, wells, overhead tanks, water meter chambers and miscellaneous household in domestic containers, for controlling mosquito menace.

## Conclusion

The present study strongly advocates the larvivore efficacy of *O. melastigma* and potency as biological control measure against mosquitoes. This fish is an Indigenous alternative to exotic larvivores. It can be recommended that breeding this fish in captive condition using *Ulothrix* as a feed supplement can meet the need of an effective larvivorous fish for mosquito control.
